# Blood Donor Deferrals for Malaria in Iran: A Five-Year Retrospective Study

**Published:** 2019

**Authors:** Ahmad MARDANI, Hossein KESHAVARZ

**Affiliations:** 1. Department of Microbiology, Blood Transfusion Research Center, High Institute for Research and Education in Transfusion Medicine, Tehran, Iran; 2. Department of Medical Parasitology and Mycology, School of Public Health, Tehran University of Medical Sciences, Tehran, Iran

**Keywords:** Malaria, Transfusion-transmitted malaria (TTM), Blood donors, Donor deferral, Iran

## Abstract

**Background:**

Malaria is one of the most important transfusion-transmitted infections (TTIs) worldwide. To prevent the occurrence of transfusion-transmitted malaria (TTM), potential blood donors with a history of malaria or travel to, or residence in, malarious areas are permanently or temporarily deferred from donating blood. The objective of the present study was to evaluate the blood donor deferrals for malaria in Iran.

**Methods:**

This descriptive cross-sectional study was conducted in the Iranian Blood Transfusion Organization (IBTO) from 21 Mar 2011 to 19 Mar 2016 (5 yr). The data were collected and extracted from IBTO comprehensive database, namely Negareh, and then recorded in a structured template form. Statistical analysis was performed using SPSS.

**Results:**

Of the 12,790,859 blood donation volunteers, 23,084 (0.18%) were deferred due to the risk of malaria. More than 90% of malaria-deferrals were because of travel to and residence in malaria endemic areas. Among the malaria-deferred volunteers, 22,139 (95.91%) were male and 945 (4.09%) were female; 2,053 (8.89%) were permanently deferred, while 21,031 (91.11%) were temporarily deferred. The highest malaria-deferral rates were observed in South Khorasan (0.82%), Razavi Khorasan (0.79%) and Yazd (0.54%) provinces, respectively.

**Conclusion:**

Given the prevalence of malaria in neighboring countries (Pakistan and Afghanistan) and several provinces of Iran and the increasing human migration and movement between malaria non-endemic and endemic areas, the malaria-deferral rate might be higher than 0.18% in Iran. Thus, the changing, as well as the precise and accurate implementation of donor selection process must be considered in all blood transfusion centers of Iran.

## Introduction

*M*alaria is the most important vector-borne infectious disease (1). Globally, 3.4 billion people are at risk of being infected with malaria. According to the latest report of the WHO, 212 million cases were reported from 91 countries and territories with 429,000 deaths in 2015 (2).

Blood transfusion is one of the potential routes of malaria transmission (3). Woolsey reported the first case of transfusion-transmitted malaria (TTM) in 1911 (4). “The occurrence of TTM in non-endemic and endemic areas of malaria is one case per four million and more than 50 cases per million units of transfused blood and blood components, respectively (5–9)”. More than 3,000 cases of TTM were reported in the world (10). The transmission of malaria via blood transfusion is a serious risk in non-immune recipients to malaria, especially if the parasite species is *Plasmodium falciparum* (5, 11–16), as the diagnosis of malaria is often missed in the recipient (11).

Malaria screening is performed in blood donors by various strategies including donor selection or screening of blood donors through interviewing, and laboratory tests (12). Since there is no reliable approved laboratory test yet available for malaria screening in blood donors (17), “the donor selection is the first, and in many countries, the only step in the prevention of TTM (18)”. In this strategy, the potential donors with a history of malaria or travel to, or residence in, malarious areas are deferred from donating blood permanently or for a certain period of time (19). These deferrals may affect the blood availability (20), although most deferred people are unlikely to be infected particularly travelers to malaria-endemic areas (12, 19, 21). The deferral rate of blood donation volunteers due to malaria risk has been reported to vary from 0.003% to 5% in different countries (9).

In 1974, the Iranian Blood Transfusion Organization (IBTO) was established in order to centralize blood and blood components supply (22, 23). The IBTO is a non-profit and national organization that currently has 31 provincial blood transfusion centers. The screening of blood donors for malaria, in endemic and non-endemic regions of Iran, is performed in the blood transfusion centers of the IBTO through interviewing by a trained physician (22–24). Based on the IBTO standard operating procedures (SOPs), volunteers who have a history of malaria are permanently deferred from blood donation. A history of travel to, or residence in, malarious areas lead to a deferral from donating blood for three years after departure and one year after their return from the endemic area, respectively. According to these instructions, the temporarily malaria-deferred volunteers after the deferral period can donate blood if they have not experienced malaria symptoms. Unfortunately, these volunteers are not investigated using laboratory (parasitological, immunological and molecular) assays.

The rate of deferred blood donation volunteers due to malaria risk has not previously been investigated in Iran and the current research is the first study conducted, to the best of our knowledge. Thus, the objective of the present study was to evaluate the blood donor deferrals for malaria in Iran.

## Materials and Methods

### Study area

**Fig. 1:**
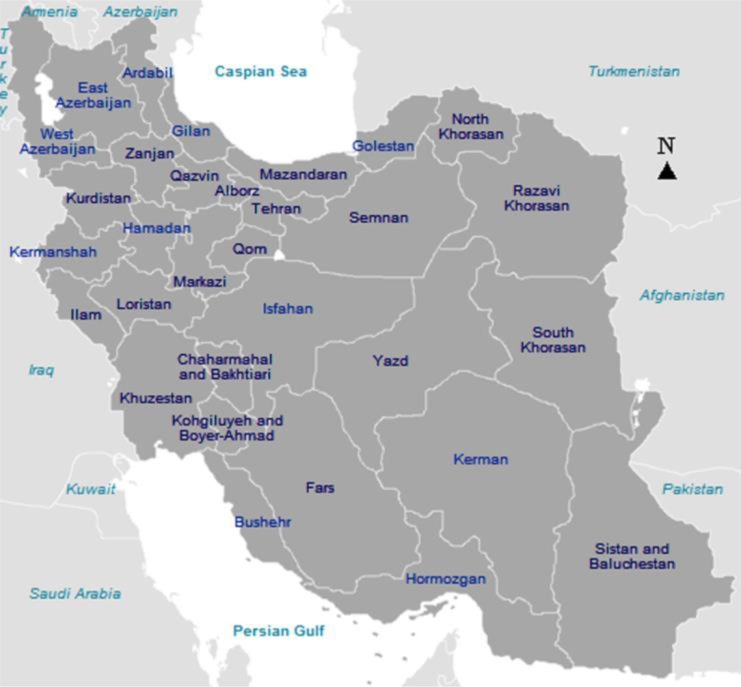
Map of 31 provinces of Iran and geographical situation of malaria endemic areas of this country namely Sistan and Baluchestan, Hormozgan and Kerman provinces, bordering Afghanistan, Pakistan, the Oman Sea and the Persian Gulf

Iran is located in the Eastern Mediterranean Region (EMR) of the WHO (2) and is subdivided into 31 provinces (Fig. 1). In Iran, malaria transmission mainly occurs in the provinces of the southeast as endemic areas with low endemicity, namely Sistan and Baluchistan, Hormozgan and Kerman (Fig. 1) (25, 26). According to the latest report of the WHO, the reported malaria cases were 1,378 in Iran during the year *2015*, of which 632 (45.86%) were imported cases. Currently, Iran is in the elimination stage of malaria (2). Although 344 cases of TTM had been reported from different provinces of Iran during the 21 years from 1963 to 1983, no case has been reported in the last three decades (10).

### Data collection and statistical analysis

In this retrospective, descriptive cross-sectional study, the comprehensive database of the IBTO, namely Negareh, was used to collect and extract the data of blood donation volunteers who presented to the blood transfusion centers of the IBTO from 21 Mar 2011 to 19 Mar 2016 (5-year period). In the IBTO, the individual characteristics and interview data, as well as the results of screening tests of blood donation volunteers are entered into the Negareh software. Therefore, this software is the best and most reliable way to access, collect and extract data of blood donors. The following data were collected and extracted from the Negareh database: total number of blood donation volunteers (the individuals who presented to the blood transfusion centers for blood donation), blood donors and deferred volunteers, as well as the total number of deferred volunteers for travelling to, or residency in, malarious areas and deferred volunteers for a past history of malaria and the blood donation volunteers’ age and sex. Finally, the extracted data were recorded in a structured template form prepared for this purpose. Statistical analysis was conducted using SPSS ver. 18.0 (Chicago, IL, USA).

## Results

During the 5-year study period, 12,790,859 blood donation volunteers were presented to the blood transfusion centers of the IBTO and 10,167,915 (79.49%) individuals successfully donated a unit of blood. Among the blood donors, 9,677,820 (95.18%) were male and 490,095 (4.82%) were female with M/F ratio 19.75:1. Of the 2,525,283 (19.74%) deferred blood donation volunteers, 23,084 (0.91%) were deferred for malaria risk. The majority of them (91.11%) were deferred because of travel to and residence in a malaria endemic area, and the remaining 2,053 (8.89%) due to a previous history of malaria. The overall rate of deferred volunteers for malaria was 0.18%. The highest malaria-deferral rate was seen in South Khorasan (0.82%), Razavi Khorasan (0.79%) and Yazd (0.54%) provinces. In contrast, the lowest malaria-deferral rate was in Ilam (0%), Ardabil (0.001%) and Kermanshah (0.002%) provinces (Table 1).

**Table 1:** Rates of blood donation and malaria-deferrals in Iran during the 5-year study period (21 Mar 2011 to 19 Mar 2016)

***Province***	Blood donation volunteers^*^, n	***Deferred volunteers, n***	***Blood donors, n***	***Malaria-deferral type***		
				**Travel and residence, n**	**History of malaria, n**	**Total, n (%)**
Alborz	359,760	98,430	261,330	152	8	160 (0.04)
Ardabil	192,144	31,397	157,735	3	0	3 (0.001)
Bushehr	226,631	55,045	171,587	653	0	653 (0.29)
Chaharmahal and Bakhtiari	162,072	33,203	128,869	225	7	232 (0.14)
East Azerbaijan	526,594	85,204	433,813	116	11	127 (0.02)
Fars	1,033,563	292,062	741,501	3,118	224	3,342 (0.32)
Gilan	436,367	57,458	377,503	106	0	106 (0.02)
Golestan	279,139	44,416	234,723	510	0	510 (0.18)
Hamadan	234,147	43,816	190,331	281	1	282 (0.12)
Hormozgan	266,129	47,020	219,109	79	56	135 (0.05)
Ilam	90,099	16,238	72,705	0	0	0 (0)
Isfahan	800,237	172,291	617,842	2,225	39	2,264 (0.28)
Kerman	468,564	86,594	372,862	661	140	801 (0.17)
Kermanshah	315,989	52,052	255,912	5	2	7 (0.002)
Khuzestan	783,718	221,203	559,015	570	75	645 (0.08)
Kohgiluyeh and Boyer-Ahmad	134,615	19,429	113,678	9	0	9 (0.006)
Kurdistan	153,630	22,172	124,961	12	0	12 (0.007)
Lorestan	241,457	47,572	193,985	5	22	27 (0.01)
Markazi	252,978	48,119	203,882	462	17	479 (0.19)
Mazandaran	777,367	124,058	637,948	627	14	641 (0.08)
North Khorasan	111,855	19,101	89,643	71	0	71 (0.06)
Qazvin	196,870	51,472	145,402	439	1	440 (0.22)
Qom	225,366	42,205	183,161	60	7	67 (0.03)
Razavi Khorasan	901,440	236,698	664,742	7,159	1	7,160 (0.79)
Semnan	215,278	31,671	172,802	308	3	311 (0.14)
Sistan and Baluchestan	417,842	68,261	347,586	497	1,381	1,878 (0.45)
South Khorasan	109,148	19,995	75,544	883	16	899 (0.82)
Tehran	2,064,411	304,469	1,759,942	245	16	261 (0.01)
West Azerbaijan	418,593	75,810	342,782	85	3	88 (0.02)
Yazd	255,380	52,740	202,640	1,370	8	1,378 (0.54)
Zanjan	139,476	25,082	114,380	95	1	96 (0.07)
Total (%)	12,790,859	2,525,283 (19.74)	10,167,915 (79.49)	21,031	2,053	23,084 (0.18)

* The individuals who presented to the blood transfusion centers for blood donation

The trend of malaria-deferred blood donation volunteers was not shown as an ascending or descending pattern over the study period.

The average age of the malaria-deferred volunteers was 34 (range 22–58) yr. Of those, 4,135 (17.91%) were in age group 25 yr or less, 15,503 (67.16%) were in group 26–45 yr, and 3,446 (14.93%) were in age group 46 yr or more. Among the malaria-deferred volunteers, 22,139 (95.91%) were male and 945 (4.09%) were female; 2,053 (8.89%) were permanently deferred, while 21,031 (91.11%) were temporarily deferred (Table 1). Of the 2,053 permanently malaria-deferred volunteers, 1,759 (85.68%) were male and 294 (14.32%) was female; 1,041 (50.71%), 635 (30.93%) and 377 (18.36%) were in age group 25 yr or less, 26–45 yr and 46 yr or more, respectively.

## Discussion

The main purpose of the blood transfusion centers is the supply of safe and adequate blood, and blood components for patients in need. Therefore, all blood donations are screened for human immunodeficiency virus (HIV), hepatitis B virus (HBV), hepatitis C virus (HCV) and syphilis (27). Several transfusion-transmitted infections (TTIs), such as malaria, babesiosis, leishmaniasis and American trypanosomiasis (Chagas disease), are not laboratory screened, and donor selection or screening of blood donors through interviewing is the only way of prevention (27, 28).

In Iran and most malaria non-endemic and endemic countries, the screening of blood donors for malaria is performed through interviewing (18, 22–24). Blood donation volunteers with a previous history of malaria or travels to, or residence in, malarious areas are deferred from blood donation permanently or for a certain period of time (19). The variety of deferral criteria is one of the most important factors that negatively impact blood availability (20). The method of blood donors screening is no different in endemic and non-endemic areas of Iran and it is carried out only through interviewing.

In the present study, the malaria-deferral rate was 0.18%, while other countries have reported different values. In Estonia 0.003%, France 0.1%–0.25%, Italy 0.1%–5%, Turkey 0.24%, Canada 0.4%, Spain 0.43%, Ireland 0.6% and the United States of America (USA) 0.75%–2.9% of blood donation volunteers were deferred for malaria risk (9, 20, 29). More than 90% of malaria-deferrals were because of travel to and residence in malaria endemic areas. Responsibility of blood donation volunteers to the issue of blood safety or their dishonesty in response to posed questions by the interviewer physician could explain the low deferral rate in this study.

The malaria-deferral rate is high in Sistan and Baluchistan (0.45%), Hormozgan (0.05%) and Kerman (0.17%) provinces as endemic areas and low in non-endemic provinces of Iran, but the highest malaria-deferral rates were observed in non-endemic provinces, including South Khorasan (0.82%), Razavi Khorasan (0.79%) and Yazd (0.54%) provinces. The higher rate might be explained by the precise and accurate implementation of donor selection and screening of blood donors in these provinces and the other non-endemic provinces such as Fars (0.32%), Bushehr (0.29%), Isfahan (0.28%) and Qazvin (0.22%) (Table 1) due to the proximity to Afghanistan, a country with high malaria endemicity (2), and endemic provinces of Iran (Fig. 1). The low rate of malaria-deferral in endemic provinces showed that either the donor selection is not well done or the individuals do not donate blood because of the related trainings with blood donation eligibility criteria in malaria-endemic areas.

The results of this study indicated that 23,084 blood donation volunteers for malaria were deferred during the 5-year study period. This rate is negligible compared to the number of blood donors (*n*=10,167,915; 79.49%) and the malaria-deferral rate has no impact on blood availability in Iran. In the USA, more than 540,000 blood donations were lost due to malaria risk from 2000 to 2006 (20).

In several European countries, the serological testing of blood donation volunteers with a history of travel to, or residence in, malarious areas has reduced the unnecessary deferrals due to malaria risk and improved the safety of blood donations (14, 17, 21, 30–33). In Turkey, 97% of malaria-deferrals were unnecessary because the serological test detecting anti-*Plasmodium* antibodies were negative (29). The unnecessary deferrals dishearten the blood donation volunteers and many of them never return to donate blood (34).

In addition to the deferrals, a number of eligible volunteers do not donate blood for various reasons. Thus, the difference is observed between the total number of blood donation volunteers and the sum of blood donors and deferred volunteers, which in this study was 0.77%.

The age of more than 85% of temporarily malaria-deferred volunteers in this study was fewer than 46 yr. These individuals can donate blood for many years without worrying if the result of an immunological or molecular assay is negative after the deferral period. Therefore, the laboratory (parasitological, immunological and molecular) assays of temporarily malaria-deferred volunteers are necessary, done in countries such as France, England and Australia (35). In Iran, unfortunately, these volunteers are not investigated using laboratory assays after the deferral period.

In this study, more than 95% of malaria-deferred volunteers were male (Table 1). Since only 4.82% of Iranian blood donors were women during the 5-year study period, this could explain the much higher rate of malaria-deferrals in men.

## Conclusion

Given the prevalence of malaria in neighboring countries (Pakistan and Afghanistan) with high endemicity and several provinces of Iran (2) and the increasing human migration and movement between malaria non-endemic and endemic areas, the malaria-deferral rate should be higher than 0.18% in Iran. Therefore, the changing of donor selection process particularly in endemic areas for the prevention of TTM, the laboratory testing of the temporarily malaria-deferred volunteers after the deferral period and the precise and accurate implementation of donor selection must be considered. In addition, the usage of a serological test detecting anti-*Plasmodium* anti-bodies or a rapid diagnostic test (RDT) for antigen detection in combination with donor selection through interviewing is essential to prevent the occurrence of TTM and reduce unnecessary deferrals prior to deferral decision in malaria non-endemic and endemic areas of Iran, respectively.
